# 5-Methyl-1*H*-indole-3-carbaldehyde

**DOI:** 10.1107/S1600536812034873

**Published:** 2012-08-11

**Authors:** Sharifah Shafiqah Ismail, Hamid Khaledi, Hapipah Mohd Ali

**Affiliations:** a Department of Chemistry, University of Malaya, 50603 Kuala Lumpur, Malaysia

## Abstract

The title mol­ecule, C_10_H_9_NO, is almost planar with an r.m.s. deviation for all non-H atoms of 0.0115 Å. In the crystal, mol­ecules are connected through N—H⋯O hydrogen bonds into chains running along [021]. The chains are further connected *via* C—H⋯π inter­actions, forming layers in the *bc* plane.

## Related literature

For the structure of 1*H*-indole-3-carbaldehyde, see: Ng (2007 ▶) and for the structure of 6-bromo-1*H*-indole-3-carbaldehyde, see: Johnson *et al.* (2009 ▶).
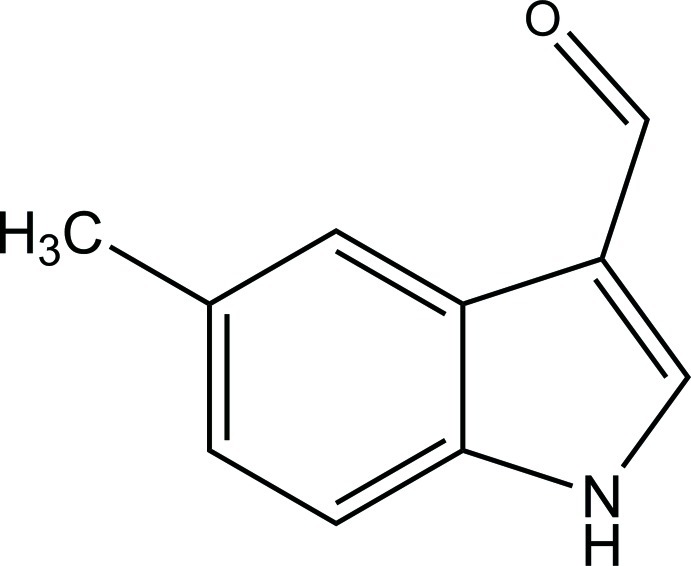


## Experimental

### 

#### Crystal data

C_10_H_9_NO
*M* *_r_* = 159.18
Orthorhombic, 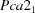

*a* = 16.9456 (19) Å
*b* = 5.7029 (6) Å
*c* = 8.6333 (9) Å
*V* = 834.31 (15) Å^3^
*Z* = 4
Mo *K*α radiation
μ = 0.08 mm^−1^
*T* = 296 K
0.47 × 0.15 × 0.05 mm

#### Data collection

Bruker APEXII CCD diffractometer
Absorption correction: multi-scan (*SADABS*; Sheldrick, 1996 ▶) *T*_min_ = 0.962, *T*_max_ = 0.996
5499 measured reflections
1147 independent reflections
717 reflections with *I* > 2σ(*I*)
*R*_int_ = 0.039

#### Refinement

*R*[*F* ^2^ > 2σ(*F* ^2^)] = 0.038
*wR*(*F* ^2^) = 0.107
*S* = 0.98
1147 reflections
113 parameters
1 restraint
H atoms treated by a mixture of independent and constrained refinement
Δρ_max_ = 0.12 e Å^−3^
Δρ_min_ = −0.14 e Å^−3^

### 

Data collection: *APEX2* (Bruker, 2007 ▶); cell refinement: *SAINT* (Bruker, 2007 ▶); data reduction: *SAINT*; program(s) used to solve structure: *SHELXS97* (Sheldrick, 2008 ▶); program(s) used to refine structure: *SHELXL97* (Sheldrick, 2008 ▶); molecular graphics: *X-SEED* (Barbour, 2001 ▶); software used to prepare material for publication: *SHELXL97* and *publCIF* (Westrip, 2010 ▶).

## Supplementary Material

Crystal structure: contains datablock(s) I, global. DOI: 10.1107/S1600536812034873/zl2498sup1.cif

Structure factors: contains datablock(s) I. DOI: 10.1107/S1600536812034873/zl2498Isup2.hkl

Additional supplementary materials:  crystallographic information; 3D view; checkCIF report

## Figures and Tables

**Table 1:** Hydrogen-bond geometry (Å, °) *Cg* is the centroid of the N1/C1/C2/C3/C8 ring.

*D*—H⋯*A*	*D*—H	H⋯*A*	*D*⋯*A*	*D*—H⋯*A*
N1—H1*N*⋯O1^i^	0.93 (3)	1.90 (3)	2.818 (3)	169 (3)
C9—H9⋯*Cg* ^ii^	0.93	2.91	3.312 (3)	107

Symmetry codes: (i) 
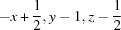
; (ii) 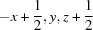.

**Fig. 1.**
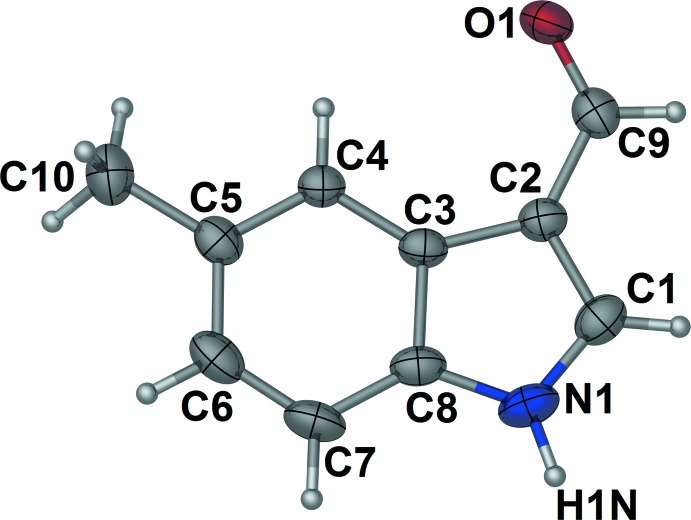
Molecular structure of the title compound showing thermal ellipsoids at the 30% prbability level. Hydrogen spheres are drawn with an arbitrary radius.

**Fig. 2.**
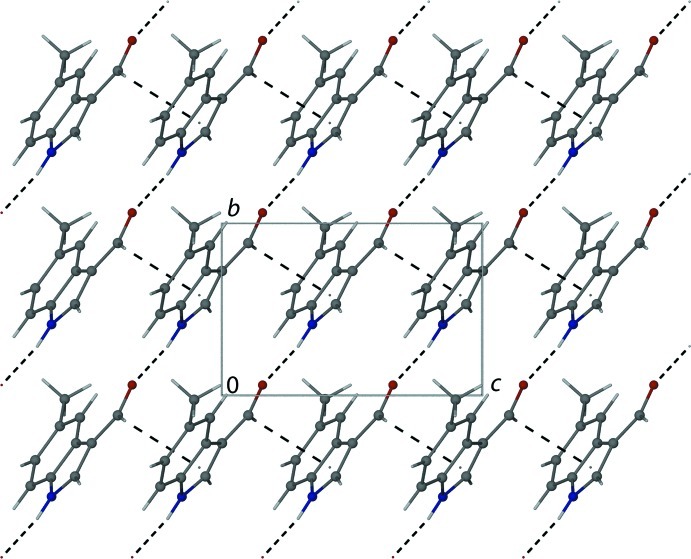
Crystal packing view looking down the a axis, thus showing the two-dimensional-supramolecular structure formed by N—H···O and C—H···π interactions (dashed lines).

## supplementary crystallographic information

### Comment

The structure of the title compound is isomorphous with that of 1*H*-indole-3-carbaldehyde (Ng, 2007). The planar molecules are connected *via* N—H···O hydrogen bonds (Table 1) into chains in the [021] direction. The chains are further linked through C—H···π interactions (Table 1) to form layers in the *bc* plane. The structure of 6-bromo-1*H*-indole-3-carbaldehyde (Johnson *et al.*, 2009) exhibits similar N—H···O bonded chains, however, further supramolecular aggregation by Br-involved interactions is observed.

### Experimental

The title crystals were obtained by slow evaporation of an ethanolic solution of the commercially available 5-methylindole-3-carboxaldehyde at room temperature.

### Refinement

The C-bound hydrogen atoms were located in calculated positions and refined in a riding mode with C—H distances of 0.93 (C*_sp2_*) and 0.96 (*C_methyl_*) Å. The N-bound H atom was found in a difference Fourier map and refined freely. For all hydrogen atoms, *U*_iso_ were set to 1.2–1.5*U*_eq_(carrier atom). In the absence of significant anomalous scattering effects Friedel pairs were merged.

### Crystal data

**Table:**

C_10_H_9_NO	*F*(000) = 336
*M**_r_* = 159.18	*D*_x_ = 1.267 Mg m^−^^3^
Orthorhombic, *P**c**a*2_1_	Mo *K*α radiation, λ = 0.71073 Å
Hall symbol: P 2c -2ac	Cell parameters from 1172 reflections
*a* = 16.9456 (19) Å	θ = 2.4–22.1°
*b* = 5.7029 (6) Å	µ = 0.08 mm^−^^1^
*c* = 8.6333 (9) Å	*T* = 296 K
*V* = 834.31 (15) Å^3^	Lath, yellow
*Z* = 4	0.47 × 0.15 × 0.05 mm

### Data collection

**Table:**

Bruker APEXII CCD diffractometer	1147 independent reflections
Radiation source: fine-focus sealed tube	717 reflections with *I* > 2σ(*I*)
Graphite monochromator	*R*_int_ = 0.039
φ and ω scans	θ_max_ = 28.8°, θ_min_ = 2.4°
Absorption correction: multi-scan (*SADABS*; Sheldrick, 1996)	*h* = −22→13
*T*_min_ = 0.962, *T*_max_ = 0.996	*k* = −7→7
5499 measured reflections	*l* = −11→11

### Refinement

**Table:**

Refinement on *F*^2^	Primary atom site location: structure-invariant direct methods
Least-squares matrix: full	Secondary atom site location: difference Fourier map
*R*[*F*^2^ > 2σ(*F*^2^)] = 0.038	Hydrogen site location: inferred from neighbouring sites
*wR*(*F*^2^) = 0.107	H atoms treated by a mixture of independent and constrained refinement
*S* = 0.98	*w* = 1/[σ^2^(*F*_o_^2^) + (0.0596*P*)^2^] where *P* = (*F*_o_^2^ + 2*F*_c_^2^)/3
1147 reflections	(Δ/σ)_max_ < 0.001
113 parameters	Δρ_max_ = 0.12 e Å^−^^3^
1 restraint	Δρ_min_ = −0.14 e Å^−^^3^

### Special details

**Table:**

Geometry. All e.s.d.'s (except the e.s.d. in the dihedral angle between two l.s. planes) are estimated using the full covariance matrix. The cell e.s.d.'s are taken into account individually in the estimation of e.s.d.'s in distances, angles and torsion angles; correlations between e.s.d.'s in cell parameters are only used when they are defined by crystal symmetry. An approximate (isotropic) treatment of cell e.s.d.'s is used for estimating e.s.d.'s involving l.s. planes.
Refinement. Refinement of *F*^2^ against ALL reflections. The weighted *R*-factor *wR* and goodness of fit *S* are based on *F*^2^, conventional *R*-factors *R* are based on *F*, with *F* set to zero for negative *F*^2^. The threshold expression of *F*^2^ > σ(*F*^2^) is used only for calculating *R*-factors(gt) *etc*. and is not relevant to the choice of reflections for refinement. *R*-factors based on *F*^2^ are statistically about twice as large as those based on *F*, and *R*- factors based on ALL data will be even larger.

### Fractional atomic coordinates and isotropic or equivalent isotropic displacement parameters (Å^2^)

**Table:**

	*x*	*y*	*z*	*U*_iso_*/*U*_eq_	
O1	0.27204 (11)	1.0610 (3)	0.6573 (2)	0.0742 (6)	
N1	0.29282 (16)	0.4126 (4)	0.3481 (3)	0.0760 (7)	
H1N	0.2766 (15)	0.285 (5)	0.290 (5)	0.091*	
C1	0.2435 (2)	0.5303 (4)	0.4384 (3)	0.0717 (8)	
H1	0.1913	0.4879	0.4563	0.086*	
C2	0.27969 (14)	0.7233 (4)	0.5018 (3)	0.0587 (6)	
C3	0.35916 (14)	0.7227 (4)	0.4414 (3)	0.0535 (6)	
C4	0.42432 (15)	0.8710 (4)	0.4561 (3)	0.0554 (6)	
H4	0.4212	1.0036	0.5185	0.066*	
C5	0.49316 (16)	0.8208 (4)	0.3782 (3)	0.0638 (7)	
C6	0.49673 (18)	0.6225 (5)	0.2839 (4)	0.0785 (8)	
H6	0.5434	0.5904	0.2311	0.094*	
C7	0.4341 (2)	0.4736 (5)	0.2661 (3)	0.0766 (8)	
H7	0.4377	0.3422	0.2026	0.092*	
C8	0.36502 (18)	0.5241 (4)	0.3453 (3)	0.0626 (7)	
C9	0.24192 (16)	0.8851 (4)	0.6026 (3)	0.0640 (6)	
H9	0.1897	0.8544	0.6292	0.077*	
C10	0.56347 (18)	0.9773 (6)	0.3966 (4)	0.0860 (10)	
H10A	0.5654	1.0866	0.3121	0.129*	
H10B	0.6107	0.8842	0.3969	0.129*	
H10C	0.5595	1.0615	0.4926	0.129*	

### Atomic displacement parameters (Å^2^)

**Table:**

	*U*^11^	*U*^22^	*U*^33^	*U*^12^	*U*^13^	*U*^23^
O1	0.0843 (15)	0.0640 (9)	0.0743 (12)	0.0106 (9)	0.0086 (10)	−0.0101 (9)
N1	0.109 (2)	0.0565 (10)	0.0622 (13)	−0.0129 (12)	−0.0146 (15)	−0.0053 (11)
C1	0.0820 (19)	0.0660 (13)	0.0671 (17)	−0.0143 (15)	−0.0109 (17)	0.0081 (14)
C2	0.0722 (18)	0.0533 (11)	0.0508 (12)	−0.0012 (11)	−0.0030 (12)	0.0033 (10)
C3	0.0668 (17)	0.0499 (10)	0.0440 (11)	0.0056 (10)	−0.0070 (11)	−0.0013 (10)
C4	0.0639 (16)	0.0528 (10)	0.0495 (12)	0.0026 (11)	−0.0052 (12)	−0.0003 (10)
C5	0.0640 (18)	0.0699 (14)	0.0575 (14)	0.0141 (12)	−0.0022 (13)	0.0073 (13)
C6	0.081 (2)	0.0869 (18)	0.0678 (16)	0.0295 (15)	0.0074 (16)	0.0032 (15)
C7	0.105 (2)	0.0657 (14)	0.0590 (16)	0.0237 (16)	0.0008 (17)	−0.0133 (12)
C8	0.089 (2)	0.0488 (10)	0.0502 (13)	0.0051 (12)	−0.0112 (14)	−0.0021 (11)
C9	0.0662 (17)	0.0683 (13)	0.0574 (14)	0.0083 (14)	0.0013 (13)	0.0124 (13)
C10	0.068 (2)	0.102 (2)	0.088 (2)	0.0008 (17)	0.0032 (16)	0.0136 (17)

### Geometric parameters (Å, º)

**Table:**

O1—C9	1.221 (3)	C4—H4	0.9300
N1—C1	1.326 (4)	C5—C6	1.395 (4)
N1—C8	1.379 (4)	C5—C10	1.497 (4)
N1—H1N	0.93 (3)	C6—C7	1.368 (4)
C1—C2	1.374 (3)	C6—H6	0.9300
C1—H1	0.9300	C7—C8	1.385 (4)
C2—C9	1.421 (3)	C7—H7	0.9300
C2—C3	1.444 (3)	C9—H9	0.9300
C3—C4	1.397 (3)	C10—H10A	0.9600
C3—C8	1.408 (3)	C10—H10B	0.9600
C4—C5	1.377 (3)	C10—H10C	0.9600
			
C1—N1—C8	109.7 (2)	C7—C6—C5	122.3 (3)
C1—N1—H1N	121.9 (18)	C7—C6—H6	118.8
C8—N1—H1N	128.1 (18)	C5—C6—H6	118.8
N1—C1—C2	111.0 (3)	C6—C7—C8	118.1 (2)
N1—C1—H1	124.5	C6—C7—H7	121.0
C2—C1—H1	124.5	C8—C7—H7	121.0
C1—C2—C9	124.3 (3)	N1—C8—C7	131.4 (2)
C1—C2—C3	105.7 (2)	N1—C8—C3	107.3 (2)
C9—C2—C3	130.0 (2)	C7—C8—C3	121.2 (3)
C4—C3—C8	119.0 (2)	O1—C9—C2	125.6 (3)
C4—C3—C2	134.7 (2)	O1—C9—H9	117.2
C8—C3—C2	106.3 (2)	C2—C9—H9	117.2
C5—C4—C3	120.0 (2)	C5—C10—H10A	109.5
C5—C4—H4	120.0	C5—C10—H10B	109.5
C3—C4—H4	120.0	H10A—C10—H10B	109.5
C4—C5—C6	119.4 (3)	C5—C10—H10C	109.5
C4—C5—C10	119.9 (2)	H10A—C10—H10C	109.5
C6—C5—C10	120.7 (3)	H10B—C10—H10C	109.5

### Hydrogen-bond geometry (Å, º)

Cg is the centroid of the N1/C1/C2/C3/C8 ring.

**Table:**

*D*—H···*A*	*D*—H	H···*A*	*D*···*A*	*D*—H···*A*
N1—H1*N*···O1^i^	0.93 (3)	1.90 (3)	2.818 (3)	169 (3)
C9—H9···*Cg*^ii^	0.93	2.91	3.312 (3)	107

Symmetry codes: (i) −*x*+1/2, *y*−1, *z*−1/2; (ii) −*x*+1/2, *y*, *z*+1/2.
